# Alexithymia Is Associated With Deficits in Visual Search for Emotional Faces in Clinical Depression

**DOI:** 10.3389/fpsyt.2021.668019

**Published:** 2021-06-29

**Authors:** Thomas Suslow, Vivien Günther, Tilman Hensch, Anette Kersting, Charlott Maria Bodenschatz

**Affiliations:** ^1^Department of Psychosomatic Medicine and Psychotherapy, University of Leipzig Medical Center, Leipzig, Germany; ^2^Department of Psychiatry and Psychotherapy, University of Leipzig Medical Center, Leipzig, Germany; ^3^Department of Psychology, IU International University of Applied Science, Erfurt, Germany

**Keywords:** alexithymia, major depressive disorder, face-in-the-crowd, emotional facial expressions, eye-tracking, visual search, anger, happiness

## Abstract

**Background:** The concept of alexithymia is characterized by difficulties identifying and describing one's emotions. Alexithymic individuals are impaired in the recognition of others' emotional facial expressions. Alexithymia is quite common in patients suffering from major depressive disorder. The face-in-the-crowd task is a visual search paradigm that assesses processing of multiple facial emotions. In the present eye-tracking study, the relationship between alexithymia and visual processing of facial emotions was examined in clinical depression.

**Materials and Methods:** Gaze behavior and manual response times of 20 alexithymic and 19 non-alexithymic depressed patients were compared in a face-in-the-crowd task. Alexithymia was empirically measured *via* the 20-item Toronto Alexithymia-Scale. Angry, happy, and neutral facial expressions of different individuals were shown as target and distractor stimuli. Our analyses of gaze behavior focused on latency to the target face, number of distractor faces fixated before fixating the target, number of target fixations, and number of distractor faces fixated after fixating the target.

**Results:** Alexithymic patients exhibited in general slower decision latencies compared to non-alexithymic patients in the face-in-the-crowd task. Patient groups did not differ in latency to target, number of target fixations, and number of distractors fixated prior to target fixation. However, after having looked at the target, alexithymic patients fixated more distractors than non-alexithymic patients, regardless of expression condition.

**Discussion:** According to our results, alexithymia goes along with impairments in visual processing of multiple facial emotions in clinical depression. Alexithymia appears to be associated with delayed manual reaction times and prolonged scanning after the first target fixation in depression, but it might have no impact on the early search phase. The observed deficits could indicate difficulties in target identification and/or decision-making when processing multiple emotional facial expressions. Impairments of alexithymic depressed patients in processing emotions in crowds of faces seem not limited to a specific affective valence. In group situations, alexithymic depressed patients might be slowed in processing interindividual differences in emotional expressions compared with non-alexithymic depressed patients. This could represent a disadvantage in understanding non-verbal communication in groups.

## Introduction

The concept of alexithymia emerged to explain symptoms of psychosomatic patients (1). It comprises the facets difficulties in identifying and describing one's feelings, a restricted imagination, and a concrete, externally oriented style of thinking (2). In the majority of studies, the self-report questionnaire 20-item Toronto Alexithymia Scale (TAS-20 (3)) has been administered to empirically measure alexithymic features. There is evidence that alexithymia occurs more frequently in men, individuals with low educational level, low socioeconomic status, and advanced age (4). It is considered a transdiagnostic, non-specific feature in many mental disorders (5). Alexithymia has been discussed as a major personality risk factor for physical illness, psychological distress, and chronic psychopathology (6, 7).

An important ability for successful interpersonal communication is the identification of emotions from facial expressions (8). Emotional information from facial expressions is decoded rapidly indicating its high biological and social relevance (9). In a crowd or group of people, salience of emotional faces becomes especially useful. In these multiple stimulus conditions, cognitive mechanisms must be activated to locate the relevant faces against the competing distractors (10). In the last decades many studies using the face-in-the-crowd paradigm have been conducted to clarify the question whether angry or happy faces are, in general, detected more efficiently. To this aim, often one emotional face has been presented in a group of neutral faces (11, 12). Some studies provided evidence for anger superiority (i.e., more efficient search for angry faces) (13, 14) whereas, other studies have observed a happiness superiority effect (i.e., more efficient search for happy faces) (15, 16). Overall, it appears difficult to draw general conclusions on the detection superiority of a specific emotion since the pattern of results observed in visual search studies could largely depend on the specific stimulus materials applied (17).

The majority of visual search experiments has based their analyses on manual reaction time and accuracy of response. Reaction time provides only a single data point per trial with which it is difficult to make precise inferences about the component operations involved in searching for target objects (18). Eye-tracking can give more direct evidence, e.g., about which objects were attended, in what sequence, for how long. Analysis of gaze behavior based on eye-tracking allows to characterize the temporal evolution of search processes in more detail. Eye movements and fixations inform primarily about overt attention processes. Covert shifts of attention are not registered by eye-tracking. Covert attention in the visual domain refers to seeing something peripherally on which the gaze is not directly focused (which is not the object of foveal vision). However, covert shifts of spatial attention are known to be involved in saccade preparation and to precede overt shifts of gaze to the target location during visual search (19). Thus, there are close functional links between covert and overt attention processes. The visual system is capable of determining a face's emotion before the face becomes the focus of attention, and facial emotions can be used by the visual system to prepare subsequent overt attention allocation (20).

In many previous face-in-the-crowd experiments, no explicit labeling or identification of emotional expressions was required (13, 21). Typically, participants were asked to decide if all faces show the same expression or if one face has an expression differing from the others. In this way, multiple emotional expressions have to be compared and differences between them have to be detected. Findings in face-in-the-crowd studies suggest that detection responses occur generally after having fixated the target (22) and that response latencies are positively associated with the number of fixations until the target is fixated (23). This means that the detection of discrepant emotional expressions at least in larger groups of stimuli requires eye-movements (overt attentional orienting) and sequential processing of expressions. In some cases, target detection (and discrimination from the distractor faces) occurs after having fixated away from the target (22). It has been argued that fast detection responses could reflect efficient guidance of the target (i.e., features of the target are already processed covertly and guide overt attention to its position) and/or efficient distractor rejection (distractor faces are skipped more frequently and/or fixated more briefly) (14, 16). Measures of eye gaze that are often used to assess visual search efficiency in face-in-the-crowd tasks comprise the time from onset of matrix display to first fixation on the target (i.e., latency to first target fixation), the number of distractors fixated before first fixation on the target and the duration of fixation time per distractor viewed before the first fixation on the target (indices of distractor processing efficiency), and the number of on-target fixations (index of target processing efficiency or deficits in disengagement) (14, 16, 22).

The personality trait alexithymia is associated with deficits in identifying others' emotional facial expressions in healthy individuals (24). There is clear evidence that these identification deficits are more pronounced under suboptimal processing conditions (e.g., when faces are presented in degraded quality or with temporal constraints (25–27)). It has been argued that alexithymia could be characterized by less efficient reading out and use of emotional information in the evaluation of facial expressions (28). According to a systematic review, alexithymic individuals' impairments in identifying emotions from facial expressions seem to be neither limited to a specific valence nor specific emotional qualities (29). That means that alexithymia has been found to be linked to deficits in identifying positive (i.e., happy) expressions as well as to deficits in identifying negative (i.e., angry, sad, and fearful) expressions. Findings from previous fMRI research with healthy individuals examining brain response to emotional faces suggest that alexithymic individuals may encode facial emotional information in general to a lesser degree at an automatic and controlled processing level (30, 31). So far, no studies have been conducted to investigate the relationship between alexithymia and visual search for emotional faces.

Alexithymic characteristics have been frequently observed in patients suffering from clinical depression (32). The prevalence of high levels of alexithymia (scoring above the upper cut-off score of the TAS-20 (33)) varies from 27% (5) to 50% (34) in depressed patients, whereas, in the general population the prevalence of high levels of alexithymia is only about 10% (35).

There exist different explanations why alexithymia goes along with increased depressive symptoms and is prevalent in depressed patients. It has been argued, e.g., that the limited ability of alexithymic individuals to regulate negative emotions may lead to chronic, undifferentiated dysphoria (36). Moreover, it has been suggested that the association between alexithymia and impaired interpersonal functioning could contribute to depression (37). Alexithymia scores in depressed patients show a relative stability over time. Several studies reported a decrease in alexithymia among depressed patients as depression severity diminishes (32, 38). However, patients' alexithymia scores in different treatment or illness phases were found to be strongly correlated, demonstrating relative stability (38, 39). The personality trait alexithymia influences course, symptoms, treatment choice, and outcome in depressed patients. It has been observed that alexithymia interferes with recovery from depression (40). Depressed patients with reduced interest in and insight into feelings are less likely to benefit from psychotherapy (41, 42). Depressed patients with high alexithymia experience a higher burden of disease and manifest higher antidepressant consumption compared with low-alexithymic patients (43).

Findings from studies in which alexithymia has not been assessed suggest that clinical depression is characterized by impairments in the identification of facial emotion across all basic emotions (e.g., fear, anger, and happiness) except sadness. However, the extent of these impairments seems to be rather small (44). Eye-tracking research has shown biased attentional preferences in the perception of emotional information in major depressive disorder under free-viewing conditions. Depressed patients allocate more attention to sad faces and less attention to happy faces compared to healthy individuals (45). Interestingly, in early studies based on the face-in-the-crowd task it was found that depressed individuals need more time to detect positive faces in crowds of neutral expressions compared to healthy controls (46, 47). Karparova et al. (48) found generally longer reaction times to positive but also to negative expressions in a face-in-the-crowd task for depressed patients. However, in three subsequent face-in-the-crowd studies, response times for happy and negative facial expressions did not vary between depressed patients and controls (49–51). In a recent fMRI study investigating cerebral reactivity to masked faces in clinically depressed patients an association between alexithymia and decreased neural response in striatal and frontal regions to negative and positive facial expressions was observed (52). Striatal and orbitofrontal areas are implicated in the detection of salient features of sensory inputs, including emotional value, and appear to contribute to automatic alerting and allocation of attention (53, 54). Thus, alexithymia seems to go along with deficits in facial emotion perception in depressed patients.

In our eye-tracking study, the relationship between alexithymia and visual processing of facial emotions in clinical depression was examined using reaction time and gaze behavior data. To our knowledge, this is the first study on attention to multiple emotional faces as a function of alexithymia using eye-tracking methodology. The analysis of patients' gaze behavior allows a rather detailed temporal exploration of attention orienting that accompanies visual search. Eye movements of alexithymic and non-alexithymic depressed patients were tracked during a face-in-the-crowd experiment in which photographs of facial expressions depicting happiness, anger, and neutral expressions were displayed. These emotional categories were examined, as previous alexithymia research has reported deficits in the identification of negative and positive facial expressions (29). To create realistic crowds of faces with some ecological validity, photographs of multiple individuals (i.e., different identities) were used in our experiment. A mixed design was implemented that included every combination of target and distractor with the three emotional expressions (angry, happy, and neutral). Angry and happy faces were examined in our investigation since previous research using the face-in-the-crowd task (in samples of healthy individuals) was focused on these two emotional expressions. Our visual search paradigm required processes of comparison and search for discrepancies between multiple facial stimuli: participants were instructed to indicate whether all stimuli are from the same category or if one (the “target” stimulus) is different from the others. Visual search efficiency differences can be explained by differential amounts of guidance provided by a target and by differences in attention allocation toward distractor stimuli (55, 56). Our depressed patients were classified as alexithymic or non-alexithymic on the basis of their TAS-20 scores (33).

We hypothesized that depressed patients with alexithymia would manifest a less efficient performance in the face-in-the-crowd task than depressed patients without alexithymia. Specifically, it was expected that alexithymic patients show slower response latencies than non-alexithymic patients in the visual search task. We focused on the analysis of four eye-gaze parameters: latency to target face (i.e., the time from onset of stimulus display to first fixation on the target), number of distractor faces fixated prior to fixating the target, number of fixations on the target, and number of distractor faces fixated after fixating the target. The last-mentioned parameter was included in our analyses because in a study like the present one on difficulties and potential delays in the perception of facial emotions it appears important to examine the processes of attention allocation after target detection. It should be noted that the parameter number of distractor faces fixated after fixating the target has rarely been used in previous research on visual search efficiency in face-in-the-crowd tasks. We also conducted group comparisons on fixation times on distractors and targets that are reported as Supplementary Material.

Latency to target (entry time of gaze on target) and number of distractor faces fixated prior to target fixation can indicate processes of attention guidance to the target face (i.e., the discrepant facial expression) (16). When a target strongly guides attention, the entry time of gaze on target should be short, few distractors are fixated, and many distractors are skipped. When a target guides attention only weakly, visual search is time-consuming, many distractors in the crowd have to be checked before the target is located. A higher number of fixations on the target indicates more attention allocation, a need for more visual information to identify the target object (57). Finally, if many distractors are fixated after the target has been visited the search and decision strategy seems to lack efficiency.

The present investigation can help to clarify which attentional or cognitive processes during visual search are impaired due to alexithymia. Alexithymic patients may manifest already deficits in early phases of visual search and scanning and look at more distractors before target fixation. However, alexithymic patients could show processing impairments only during or after target fixation. That is, they might exhibit a higher number of fixations on target or a higher number of distractor fixations after target fixation than non-alexithymic patients. Post-target detection deficits in the face-in-the-crowd task could suggest difficulties in the processing of similarities and discrepancies between facial expressions and the integration of collected information into a decision. The present task enables to explore whether alexithymic processing deficits concern perception of angry faces, happy faces or both types of expressions.

## Materials and Methods

### Participants

Patients from the Department of Psychosomatic Medicine and Psychotherapy at the University of Leipzig participated in the study. They fulfilled the criteria for a DSM-IV diagnosis of major depressive disorder as assessed by the Structured Clinical Interview for the DSM-IV Axis I (58). Exclusion criteria were other past or present bipolar, schizophrenia or psychotic disorders, abuse of alcohol or other substances within the past 6 months, medical diagnoses associated with neurocognitive impairments, treatment with sedatives, or antipsychotics as well as the wearing of eyeglasses or contact lenses. The 20-item Toronto-Alexithymia-Scale (TAS-20 (3); German version (59)) was administered to classify patients as alexithymic and non-alexithymic. The criteria proposed by Bagby and Taylor (33) were applied to define alexithymia and non-alexithymia. Patients scoring ≥61 were considered alexithymic (*n* = 20) and those scoring ≤ 51 were considered non-alexithymic (*n* = 19). Fifty-four percent of the sample were medicated with antidepressants (*N* = 21).

Our study was approved by the ethics committee at the University of Leipzig, Medical Faculty, and in accordance with the Declaration of Helsinki. We obtained informed consent from all patients prior to inclusion and all patients were financially compensated.

### Psychological Measures

The Montgomery-Asberg Depression Rating Scale (MADRS (60)), an interviewer-administered scale, was applied to assess severity of depression. The BDI-II (61) was administered to assess severity of depressive symptoms by self-report. The State-Trait Anxiety Inventory (STAI (62)) was used in its state form to assess anxious feelings at the time of testing. The Trail Making Test Part B (TMT-B (63)) was given to the patients to control for possible differences between groups in visual search speed and cognitive flexibility.

### Stimuli and Face-in-the-Crowd Task

Face stimuli comprised 24 photographs of eight individuals (four women, four men) selected from the Lifespan Database of Adult Emotional Facial Stimuli (64). Stimuli comprised three types of emotional expressions (angry, happy, and neutral faces). All photographs were processed to replace background features and to limit each facial expression to head and neck. All faces were in the same frontal orientation, similar in size and gray scaled.

In each trial, eight photographs arranged in a circle were shown simultaneously against a black background. All stimulus matrices were viewed at a distance of 70 cm with a visual angle of ~22.9° × 21.6° (height × width). Each face subtended a visual angle of 6° × 3.9° (height × width). The centers of adjacent faces were located at the same distance (6.5°). Within the same trial, positions were randomly assigned, and identities did not repeat. One-third of the trials were target absent (*n* = 24), i.e., composed of only one expression condition (e.g., all faces depicted angry expressions). Two-thirds were target-present trials (*n* = 48), showing one face from an expression condition and seven faces from a discrepant condition (e.g., one angry face among seven neutral faces). All target/distractor combinations were utilized (i.e., angry target happy distractors, angry target neutral distractors, happy target angry distractors, happy target neutral distractors, neutral target happy distractors, and neutral target angry distractors). In the target-present trials, each expression condition appeared once in each of the eight possible positions, resulting in eight trials for each target-distractor combination. For each participant, the order of trials was randomized.

### Eye-Tracking Procedure

Patients were tested individually by an experienced experimenter. Camera adjustments were made to best capture eyes of patients. A nine-point grid was used for calibration. Calibration was repeated in case the deviation exceeded x/y 0.7°.

Each trial began with a fixation cross, displayed until a fixation of 1,000 ms. Then, face stimuli were shown until response or, in case of no response, for 5,000 ms. Subjects were instructed on the computer screen that they would see a series of faces arranged in a circle. Their task was to press the response key quickly whenever one of the faces differed in its expression from the others.

### Eye Movement Apparatus and Parameter

SMIs Experiment Center software was applied to display stimuli and to synchronize with recorded eye movements. Pictures were presented on a 22-inch TFT widescreen monitor (resolution: 1680 × 1050) running with an SMI-customized Dell laptop. Viewing behavior was continuously registered with an IView X RED250 remote system by SMI, an infrared video-based eye-tracker recording eye movements every 4 ms (250 Hz) with a gaze position accuracy of 0.4°.

Gaze data were analyzed using a velocity-based algorithm with a minimum saccade duration of 22 ms, a peak velocity threshold of 40°/s, and a minimum fixation duration of 100 ms. We used BeGaze 3.0 (SMI, Teltow) to define eight areas of interest (AOIs) in each trial corresponding to each of the eight face expressions.

Manual response times were measured, i.e., the time between picture onset and key press. The rates of correct responses and non-responses were computed for all stimulus conditions. Four main measures of gaze behavior were used. First, we calculated the latency to target face or entry time on target (i.e., the time from onset of stimulus display to first fixation on the target). Second, we analyzed whether patient groups differed concerning attention guidance to the target face. Thus, we determined the number of distractor faces fixated prior to fixating the target. When a target strongly attracts attention, only few distractors should be fixated, and many distractor stimuli should be neglected. In case a target stimulus guides attention only weakly, many distractors in a group of stimuli must be analyzed before the target is finally identified (16). Third, we wanted to investigate whether patient groups differed regarding target processing. Therefore, we analyzed the mean number of fixations on the targets. Fourth, we determined the number of distractor faces fixated after fixating the target. If many distractors are analyzed after the target has been visited search and decision-making seems to lack efficiency.

Additional analyses of gaze behavior were conducted using fixation duration parameters to determine stimulus processing of targets and distractors. Mean fixation times per distractor face before and after fixating the target were calculated, respectively. Mean fixation time on targets were also analyzed. For the sake of brevity, only the main findings of these analyses based on fixation duration will be included in this article. The relevant fixation data and statistical results are described in more detail in the Supplementary Material.

The analyses of reaction time and eye-movement data focus on the target present trials with correct responses. The rate of correct responses across all target present conditions was 0.98 (SD: 0.03). Reaction times and eye-movement measures were analyzed using 6 (condition) × 2 (group) mixed ANOVAs. Analysis of covariance (ANCOVA) was used to control for covariates of interest when looking at group differences in test performance and gaze behavior. One-sample *t*-tests were administered as *post-hoc* tests to assess differences in decision performance or gaze behavior between face conditions in the total sample. The Shapiro-Wilk test was used to examine if reaction-time and eye-movement variables were normally distributed. In case of (partial) violation of normality for reaction-time and eye-movement data, Mann-Whitney *U*-tests were calculated to compare performance between groups. Two-sample *t*-tests and Chi^2^-tests were applied to identify group differences in sociodemographic, clinical, and psychological characteristics and test performance.

### General Procedure

The experiment took place at the Department of Psychosomatic Medicine and Psychotherapy at the University of Leipzig. After the clinical screening procedure described above, patients were invited to the experimental session individually. The experiment was conducted in a sound-attenuated room shielded from sunlight. Ceiling lighting produced stable illuminance conditions. After the eye-tracking experiment, participants completed the BDI-II, the state version of the STAI, and the TMT-B.

## Results

### Sociodemographic, Clinical, and Psychological Characteristics

Study groups did not differ in age, gender distribution, state anxiety, visual search speed (TMT-B), interviewer-rated depression (MADRS), illness onset (years since first depressive episode), and number of experienced depressive episodes (see Table 1 for details). However, alexithymic patients had a lower level of education, *t* (37) = −2.94, *p* < 0.01, reported more depressive symptoms (BDI), *t* (37) = 2.56, *p* < 0.05, and took more frequently antidepressants, Chi^2^(1) = 4.31, *p* < 0.05 (see Table 1). According to both, MADRS and BDI, patients suffered from moderate levels of depressive symptoms at time of testing.

**Table 1:** Demographic, clinical, and questionnaire characteristics of alexithymic and non-alexithymic depressed patients [means and SD (in brackets) or frequency values].

**Variable**	**Alexithymic patients**	**Non-alexithymic patients**	***p***
Age	28.80 (7.41)	30.11 (6.69)	n.s.
Gender (f/m)	13/7	13/6	n.s.
Level of education^a^	3.15 (1.42)	4.42 (1.26)	<0.01^*^
Years since first depressive episode	7.85 (6.19)	9.11 (4.75)	n.s.
Number of episodes	6.60 (7.81)	6.32 (6.73)	n.s.
Antidepressant medication (yes/no)	14/6	7/12	<0.05^*^
TMT-B (seconds)	62.56 (18.36)	58.96 (23.12)	n.s.
BDI-II (sum score)	25.50 (6.60)	20.47 (5.60)	<0.05^*^
MADRS (sum score)	25.35 (4.58)	23.18 (5.32)	n.s.
STAI-S (item score)	2.29 (0.42)	2.32 (0.55)	n.s.
TAS-20 (sum score)	66.70 (5.56)	44.32 (5.82)	<0.001^*^

* *Significant differences between groups according to independent samples t-tests or χ^2^ tests*.

a *Coding of level of education: 1 = 9th grade, 2 = 10th grade, 3 = 11th grade, 4 = 12th grade, 5 = University bachelor degree, 6 = University master degree*.

*TMT-B, Trail Making Test Part B; BDI-II, Beck Depression Inventory II; MADRS, Montgomery-Åsberg Depression Rating Scale; STAI-S, State-Trait Anxiety Inventory – state version; TAS-20, 20-Item Toronto-Alexithymia Scale*.

### Manual Response Data

Rates of correct responses and non-responses were high for both study groups (see Table 2). The results of a 6 (condition) × 2 (group) mixed ANOVA on correct response rates in target-present trials indicate a significant main effect of condition *F*_(5, 185)_ = 5.93, *p* < 0.001, $\eta_{p}^{2}$ = 0.14, but no main effect of group, *F*_(1, 37)_ = 1.23, *p* = 0.27, and no interaction effect, *F*_(5, 185)_ = 1.15, *p* = 0.34. The experimental conditions “angry target neutral distractors” and “neutral target angry distractors” had overall the lowest rates of correct responses. The results of one-sample *t*-tests show that correct response rate for “neutral target angry distractors” was lower than that in the conditions “happy target angry distractors,” “angry target happy distractors,” “happy target neutral distractors” and “ neutral target happy distractors” (*p*s < 0.05). Similarly, correct response rate for “angry target neutral distractors” was lower than that in the conditions “angry target happy distractors,” “happy target neutral distractors” and “neutral target happy distractors” (*p*s < 0.05).

**Table 2:** Rate of correct non-responses/responses as a function of alexithymia, emotional quality of target, and distractor face and target absence/presence [means and SD (in brackets)].

**Variable**	**Alexithymic patients**	**Non-alexithymic patients**
All angry faces	0.94 (0.09)	0.99 (0.06)
All happy faces	0.99 (0.03)	0.99 (0.03)
All neutral faces	0.98 (0.05)	0.97 (0.05)
Angry target happy distractors	0.99 (0.03)	0.99 (0.04)
Angry target neutral distractors	0.94 (0.08)	0.97 (0.06)
Happy target angry distractors	0.96 (0.08)	1.0 (0.0)
Happy target neutral distractors	0.99 (0.04)	0.99 (0.06)
Neutral target angry distractors	0.94 (0.09)	0.95 (0.08)
Neutral target happy distractors	1.0 (0.0)	0.99 (0.03)

According to two-sample *t*-tests alexithymic and non-alexithymic patients did not differ on trials with only neutral or only happy faces concerning rate of correct non-responses. However, alexithymic patients showed fewer correct non-responses than non-alexithymic patients for trials consisting only of angry faces, *t* (37) = −2.10, *p* < 0.05. Since rates of correct non-responses and responses were not normally distributed for all conditions in both groups, additional non-parametric analyses were calculated. According to Mann-Whitney *U*-tests, alexithymic patients had fewer correct responses than non-alexithymic patients in trials consisting only of angry faces, (*U* = 127, *p* < 0.05). Moreover, alexithymic patients showed fewer correct responses than non-alexithymic patients in trials with a happy target in angry distractors, (*U* = 152, *p* < 0.05).

**Figure 1:**
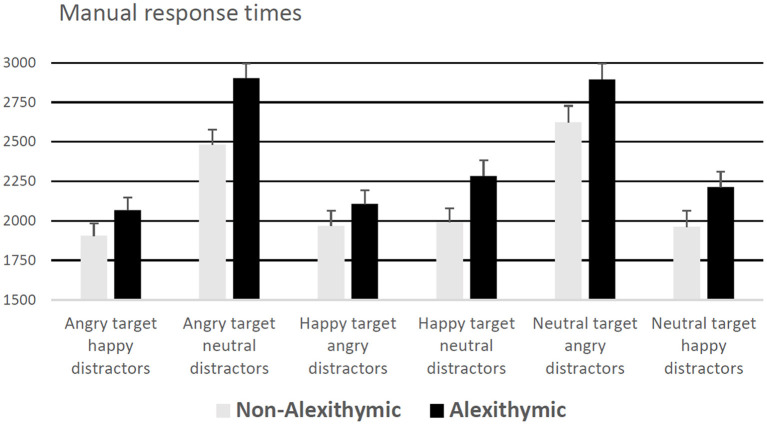
Manual response times (for correct responses) in ms as a function of alexithymia and emotional quality of target and distractor face (error bars represent standard error).

A 6 (condition) × 2 (group) mixed ANOVA on response latencies revealed a significant effect of condition, *F*_(5, 185)_ = 68.13, *p* < 0.001, $\eta_{p}^{2}$ = 0.65, and a significant effect of group, *F*_(1, 37)_ = 5.73, *p* <0.05, $\eta_{p}^{2}$ = 0.13, but no interaction effect, *F*_(5, 185)_ = 1.46, *p* = 0.21. Alexithymic patients exhibited in general slower decision latencies compared to non-alexithymic patients (see Figure 1). Independent of study group, participants responded slowest in the conditions “neutral target angry distractors” and “angry target neutral distractors” (see Figure 1). According to one-sample *t*-tests, response latencies in the condition “neutral target angry distractors” were significantly higher than those in the conditions “happy target angry distractors,” “angry target happy distractors,” “happy target neutral distractors” and “neutral target happy distractors” (*p*s < 0.05). Moreover, response latencies in the trials “angry target neutral distractors” were higher than those in the trials “angry target happy distractors,” “happy target neutral distractors” and “neutral target happy distractors” (*p*s < 0.05).

In addition, an ANCOVA was performed entering level of education, reported depressive symptoms (BDI), antidepressant use, and sex as covariates. The ANCOVA results showed that the covariates did not have significant effects on the dependent variable, whereas, the effect of group remained significant, *F*_(1, 33)_ = 5.36, *p* < 0.05, $\eta_{p}^{2}$ = 0.14.

### Eye-Movement Data

#### Latency to Target

**Figure 2:**
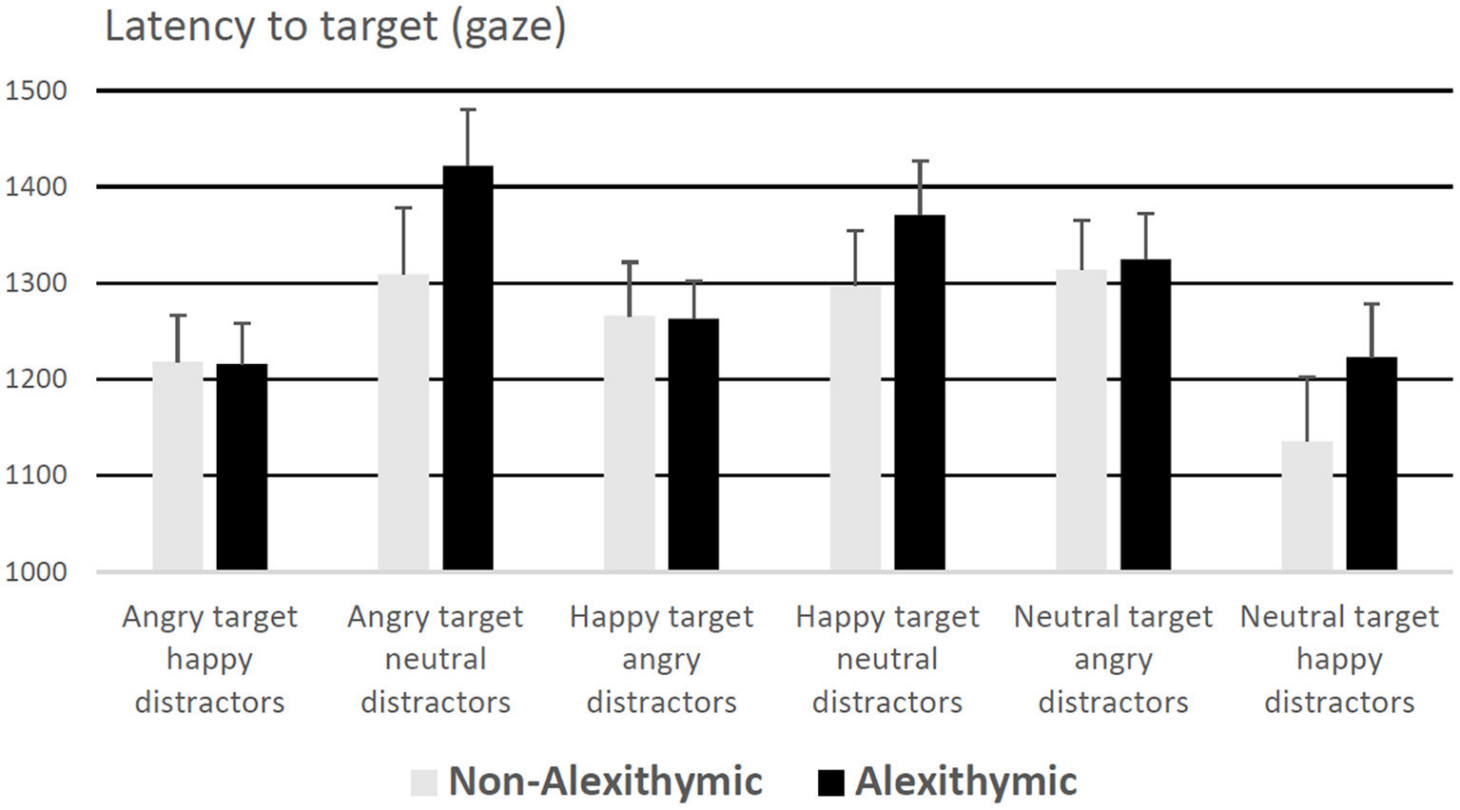
Latency to target (for correct responses) in ms as a function of alexithymia and emotional quality of target and distractor face. Error bars represent standard error.

A 6 (condition) × 2 (group) mixed ANOVA on entry times of gaze on target revealed a main effect of condition, *F*_(5, 185)_ = 5.41, *p* < 0.001, $\eta_{p}^{2}$ = 0.13, no effect of group *F*_(1, 37)_ = 0.80, *p* = 0.38, and no interaction effect, *F*_(5, 185)_ = 0.67, *p* = 0.65. Participants' orientation of gaze to the target face was slowest in the conditions “neutral target angry distractors” and “angry target neutral distractors,” regardless of study group (see Figure 2 for details).

#### Number of Distractor Faces Fixated Prior to Fixating the Target

Analyses revealed a main effect of condition, *F*_(5, 185)_ = 3.41, *p* < 0.01, $\eta_{p}^{2}$ = 0.08, but no main effect of group *F*_(1, 37)_ = 0.04, *p* = 0.85, and no interaction effect, *F*_(5, 185)_ = 1.22, *p* = 0.30. Independent of group, participants fixated more distractor faces in the conditions “angry target neutral distractors” and “neutral target angry distractors” followed by “happy target neutral distractors,” and “angry target happy distractors (see Table 3). Participants fixated fewer distractors in the conditions “happy target angry distractors” and “neutral target happy distractors.”

**Table 3:** Fixation of distractor faces (number of faces) before target fixation as a function of alexithymia and emotional quality of target and distractor face [means and SD (in brackets)].

**Variable**	**Alexithymic patients**	**Non-alexithymic patients**
Number of fixated happy distractors before fixating angry target	3.46 (0.72)	3.60 (0.63)
Number of fixated neutral distractors before fixating angry target	3.87 (0.75)	3.80 (0.87)
Number of fixated angry distractors before fixating happy target	3.38 (0.46)	3.41 (0.79)
Number of fixated neutral distractors before fixating happy target	3.61 (0.73)	3.68 (0.54)
Number of fixated angry distractors before fixating neutral target	3.63 (0.71)	3.96 (0.75)
Number of fixated happy distractors before fixating neutral target	3.55 (0.84)	3.17 (0.68)

#### Number of Fixations on the Target

ANOVA revealed a main effect of condition, *F*_(5, 185)_ = 32.71, *p* < 0.001, $\eta_{p}^{2}$ = 0.47, but no main effect of group *F*_(1, 37)_ = 0.43, *p* = 0.51, and no interaction effect, *F*_(5, 185)_ = 1.77, *p* = 0.12. Study participants fixated in general the target face longest in the conditions “neutral target angry distractors” followed by “angry target neutral distractors” (see Table 4 for details).

**Table 4:** Number of fixations on target as a function of alexithymia and emotional quality of target and distractor face [means and SD (in brackets)].

**Variable**	**Alexithymic patients**	**Non-alexithymic patients**
Number of fixations on angry target in happy distractors	1.46 (0.27)	1.57 (0.32)
Number of fixations on angry target in neutral distractors	2.03 (0.39)	1.76 (0.31)
Number of fixations on happy target in angry distractors	1.59 (0.30)	1.51 (0.36)
Number of fixations on happy target in neutral distractors	1.46 (0.37)	1.43 (0.26)
Number of fixations on neutral target in angry distractors	2.16 (0.48)	2.11 (0.45)
Number of fixations on neutral target in happy distractors	1.65 (0.35)	1.64 (0.46)

#### Number of Distractor Faces Fixated After Fixating the Target

A 6 × 2 ANOVA yielded a significant main effect of condition, *F*_(5, 185)_ = 28.42, *p* < 0.001, $\eta_{p}^{2}$ = 0.43, and a significant main effect of group, *F*_(1, 37)_ = 6.79, *p* < 0.05, $\eta_{p}^{2}$ = 0.15. No interaction effect was observed, *F*_(5, 185)_ = 0.57, *p* = 0.72. Alexithymic patients fixated more distractors after target fixation than non-alexithymic patients regardless of face condition (see Table 5). Independent of group, participants fixated more distractor faces after fixating the target in the conditions “angry target neutral distractors” and “neutral target angry distractors” than in the other experimental conditions.

**Table 5:** Fixation of distractor faces (number of faces) after target fixation as a function of alexithymia and emotional quality of target and distractor face [means and SD (in brackets)].

**Variable**	**Alexithymic patients**	**Non-alexithymic patients**
Number of fixated happy distractors after fixating angry target	1.30 (0.78)	0.71 (0.69)
Number of fixated neutral distractors after fixating angry target	2.90 (1.07)	2.12 (1.02)
Number of fixated angry distractors after fixating happy target	1.45 (1.02)	1.07 (0.96)
Number of fixated neutral distractors after fixating happy target	1.69 (1.01)	1.07 (0.65)
Number of fixated angry distractors after fixating neutral target	2.89 (1.34)	2.09 (1.22)
Number of fixated happy distractors after fixating neutral target	1.56 (1.14)	1.19 (0.79)

Data for “number of distractor faces fixated after fixating the target” were in the majority of conditions normally distributed (8 out of 12). Only in case of the conditions “angry target happy distractors” (for the non-alexithymic group), “happy target angry distractors” (for both groups) and “neutral target happy distractors” (for the non-alexithymic group) data did not show a normal distribution. According to the results of additional Mann-Whitney *U*-tests, alexithymic patients fixated more distractors after target fixation than non-alexithymic patients in the conditions “angry target happy distractors” (*U* = 101.5, *p* < 0.05) and “angry target in neutral distractors (*U* = 112, *p* < 0.05). Moreover, they tended to fixate more distractors after fixation of the target than non-alexithymic patients in the conditions “happy target neutral distractors” (*U* = 128, *p* <0.10) and “neutral target angry distractors” (*U* = 126, *p* < 0.10). Number of fixated distractors after target fixation did not differ between groups for “happy target angry distractors” (*U* = 136, *p* = 0.13) and “neutral target happy distractors” (*U* = 163.5, *p* = 0.45). Most importantly, the number of distractor faces fixated after target fixation across all conditions differed between study groups (*U* = 107, *p* < 0.05): alexithymic patients fixated overall more distractors after fixating the target than non-alexithymic patients.

An ANCOVA was calculated with level of education, reported depressive symptoms (BDI), use of antidepressants, and sex as covariates. The results suggest that out of the covariates only education level had a significant effect on the dependent variable, *F*_(1, 33)_ = 6.04, *p* < 0.05, $\eta_{p}^{2}$ = 0.15: higher level of education was found to be associated with fewer fixated distractors after target fixation. The effect of group remained significant, *F*_(1, 33)_ = 7.87, *p* < 0.01, $\eta_{p}^{2}$ = 0.19.

### Supplemental Analyses: Fixation Duration on Targets and Distractors

An ANOVA conducted on fixation time on targets suggests no difference between study groups or interaction effect. The analyses of mean fixation times per distractor face before fixating the target also revealed no difference between study groups or interaction effect. According to an ANOVA and additional ANCOVA controlling for education level, depressive symptoms, use of antidepressants, and sex alexithymic patients fixated distractor faces longer than non-alexithymic patients after target fixation, regardless of face quality (see Supplementary Material for details).

## Discussion

In our study, we investigated the relationship between alexithymia and visual processing of facial emotions in clinical depression. To this aim, we analyzed reaction times and gaze behavior in a face-in-the-crowd task. The concept of alexithymia refers to difficulties in identifying, describing one's feelings and an external orientation of thought (2), and is considered a major risk factor for physical and mental illness (6, 7). This is the first study on attention to multiple emotional faces as a function of alexithymia using eye-tracking methodology. Our visual search task required processes of comparison and search for discrepancies between multiple facial expressions of different individuals. Our task did not ask participants to explicitly identify or label facial emotions so that it appears plausible to assume that the processes of categorization and comparison operated primarily implicitly. Two groups of patients suffering from major depression were compared that differed substantially concerning their alexithymia scores. In our study, alexithymia was empirically measured *via* the internationally widely used 20-item Toronto Alexithymia-Scale (65, 66). Research using the TAS-20 has demonstrated adequate levels of convergent and concurrent validity of this self-report instrument (3). One patient group showed clinically relevant alexithymic characteristics whereas, the other patient group included non-alexithymic individuals according to the criteria of Bagby and Taylor (33).

There were no differences between our study groups with regard to age, sex, illness onset, number of illness episodes, general visual search speed (TMT-B), state anxiety, and interviewer-rated depression. However, alexithymic patients took more frequently antidepressants, reported more depressive symptoms, and had a lower level of education than non-alexithymic patients. Therefore, these variables (and sex) were taken into consideration as covariates in the group comparisons. Associations of alexithymia with increased antidepressant consumption (67), heightened psychological distress (43), and lower education (4) have been observed previously. Depressed patients with alexithymia are known to often notice and report physical symptoms (68). Given alexithymic patients' tendency to describe somatic symptoms physicians might be more inclined to treat these patients with medications.

According to our reaction time findings, alexithymic depressed patients manifested in general longer decision latencies in the face-in-the-crowd task compared to non-alexithymic depressed patients. Thus, patients with alexithymia were slower in the visual search for and comparison between emotional facial expressions than patients without alexithymia. The present findings corroborate our hypothesis that patients with alexithymia manifest a less efficient performance in the face-in-the-crowd task than patients without alexithymia. In our study, rates of correct responses (and non-responses) were high for both study groups suggesting that participants understood and attentively performed the task. For target present trials, correct response rates did not differ between patient groups (except for trials with a happy target in angry distractors: here alexithymic patients gave fewer correct responses than non-alexithymic patients). Moreover, patient groups showed a similar rate of correct answers on trials with only neutral or only happy facial expressions. However, alexithymic patients made fewer correct decisions than non-alexithymic patients when only angry faces were displayed. Thus, we found some evidence for deficits in comparing threatening facial expressions in alexithymic depressed patients.

As reaction times in visual search tasks provide only a summary or final snapshot of attention processes it was a central goal of our study to decompose attention allocation into different components by analyzing gaze behavior over time. According to our results, patient groups differed neither in latency to target (i.e., the time from stimulus onset to first fixation of the discrepant facial expression in a crowd) nor in the number of fixations on target. Therefore, it appears that alexithymic patients were on the target faces as quickly as non-alexithymic patients and they fixated them as frequently as non-alexithymic ones, regardless of whether targets were emotional or non-emotional. Moreover, there were no differences between patient groups for number of distractors fixated prior to target fixation. In our sample, patients fixated on average three to four distractor faces before their gaze was directed to the target. In sum, it can be concluded from these eye-tracking data that no discrepancies were found between alexithymic and non-alexithymic depressed patients in early gaze behavior, i.e., from stimulus onset to processing of the target face. Hence, it seems that alexithymia is not associated with abnormalities in processes of attention guidance to the target face. Alexithymic patients do not have to check more distractor faces before the target is located compared to non-alexithymic patients.

The results are different when considering patients' gaze behavior after target detection. After having looked at the target face, alexithymic patients fixated more distractors than non-alexithymic patients regardless of face condition. This pattern of findings is confirmed by the results of our supplemental analyses concerning fixation duration. That is, after fixating the target alexithymic patients looked at distractor faces longer than non-alexithymic patients but there were no group differences in fixation time on distractors before target fixation and fixation time on target. The present data could indicate processing deficits only *after* target fixation in alexithymic patients. However, it cannot be excluded that a less efficient processing and identification of the target face expression has led to an increased requirement in alexithymic patients to look more often at further (distractor) faces before they came to a correct decision (i.e., that one of the faces differs in its expression from the others). At this point, it must be emphasized that the arguments presented here to explain the observed group differences have a rather speculative and tentative character and that further research and experimental evidence are needed for solid conclusions. It can also be argued that if distractor faces are fixated after the target face has been visited decision-making lacks efficiency (57). The observed deficits after target detection might suggest difficulties in the processing of similarities and discrepancies, and the integration of the gathered information into a decision. It is possible that alexithymic patients have specific problems in comparing emotional (and neutral) faces and deciding whether the expressions belong to a single category or not. The alexithymic patients might feel uncertain about the perceived expressions and could need more information before making a final decision. Lorey et al. (69) demonstrated in an experiment with video scenes of human interactions that people with high alexithymia are less confident about assessing others' emotions than those with low alexithymia. In their study, participants had to perceive emotions depicted in point-light displays and assess the confidence in these perceptions. Interestingly, people with high alexithymia were significantly less confident about their decisions but did not differ from people with low alexithymia in the valence of their ratings.

However, in our view it cannot be excluded that although alexithymic patients did not differ from non-alexithymic patients in initial distractor fixations and target fixations (regarding duration and number of fixations) they might have still processed and encoded less facial emotional information per fixation in the early phase of visual search. In general, increased fixation duration may reflect or enable more attention to and deepened processing of the fixated object (70). Consistently, it has been observed that fixation frequency during visual exploration of pictures is positively related to subsequent recall performance (71). If alexithymic patients have deficits in encoding emotional information they could need extra time during visual search for gathering more information on the composition of the crowd of faces. Findings from previous neuroimaging research on the perception of (single) emotional facial expressions show that alexithymia goes along with reduced neural response in various parts of the brain in healthy individuals (31) and depressed patients (52). It has been argued that alexithymic individuals could manifest impairments in the perceptual encoding of emotional information at an automatic processing level (30). Yet, when looking at the specific abnormalities shown by our alexithymia patients in late (but not early) gaze behavior it appears likely that their impairments in the face-in-the-crowd task are more due to difficulties in comparing different emotional facial expressions, integrating the perceived information, and coming to a decision on dissimilarity of expressions than to general encoding deficits. Similarly, findings from a sequential affective priming study (28) indicate that alexithymic individuals could be less efficient in the use of emotional facial information when assessing subsequently shown neutral facial expressions.

In our visual search study, we investigated attention to happy and angry faces, as previous alexithymia research has revealed impairments in the identification of positive and negative facial expressions (29). The present results are consistent with the idea that alexithymic individuals' impairments in processing emotions in facial expressions are not limited to a specific affective valence. The alexithymic processing deficits seem to concern both types of expressions presented in our experiment, happy, and angry faces. Our results suggest a general, emotion-unspecific visual processing deficit in depressed patients with alexithymia.

A point worthy of note is that independent of patient group an effect of valence or valence combination was observed in our face-in-the-crowd task. Patients performed worst in face conditions where an angry target was combined with a neutral crowd or a neutral target with an angry crowd. Here, patients required substantially more time to respond and to find the target, they made more fixations on the crowd faces prior to target fixation, and they fixated the target face longer in comparison with other expression conditions. This pattern of results shows that it was much more difficult for our patients to find the target when angry and neutral faces were combined compared to other combinations of expressions. Most likely, they had difficulties to differentiate between these two categories of expression. Categorization of stimuli as target vs. distractor should take more time when distractor stimuli and target are similar to each other. The present findings indicating faster processing of happy expressions in crowds of faces are consistent with results from other research indicating a superiority effect for happy faces (14, 15). However, as mentioned earlier, it appears difficult to draw general conclusions about advantages for processing a specific facial emotion in groups of faces as some studies have reported a superiority effect for angry expressions (16, 17). It seems that the results observed in visual search for emotion faces could largely depend on the specific stimulus set applied (17).

Interestingly, even though, the processing of crowds comprising angry and neutral expressions was more difficult in our study than the processing of crowds with happy faces, there was no evidence that alexithymic patients' processing deficits were more pronounced in or limited to the most challenging task condition.

Based on the present findings, we suggest that future investigations of emotion processing in clinical depression obtain measures of alexithymia in order to determine whether any deficits or abnormalities observed are caused by depression or alexithymia. The control of alexithymia in research on emotion perception in depression seems to be of importance not least because it is fairly common in depressed patients (5, 34). Presence of alexithymia may define a subgroup of depressed patients who exhibit specific impairments in the perception of others' emotions. Interestingly, as there are elevated rates of alexithymia and emotion processing dysfunctions in a number of mental disorders (e.g., autism, substance abuse, and eating disorders) it has been suggested to assess routinely the role of alexithymia in emotion perception across different disorders (72).

According to our results, alexithymic depressed patients could be slow in the identification of discrepancies between facial emotions expressed by different individuals. Thus, in group situations alexithymic patients might be slower in noting that the emotional expression of a person deviates from the emotions expressed by the others compared with non-alexithymic patients. This could represent a disadvantage in comprehending emotional group dynamics, especially in case emotional responses of group members change fast and frequently. Alexithymic individuals' deficient emotion identification ability could be an important factor contributing to their difficulties in using interpersonal communication with others to manage distress (73). Alexithymia itself should become more often the target for psychological interventions. Findings from treatment studies suggest that it might be partly modifiable and improvements in alexithymia can be accompanied by improvements in other domains of functioning such as interpersonal abilities (74, 75). Recently, a promising psychological intervention method to reduce alexithymia has been proposed that combines psychoeducation with a smartphone-based emotion recognition skills training (76).

Limitations of our study include small sample sizes and the sole reliance on self-report for measuring alexithymia. The categorical research approach that we employed to examine the potential effects of alexithymia on visual emotion processing can be viewed critically. The comparison of extreme groups leads to the neglect of in-between participants. In our study, this neglect concerns individuals with TAS-20 scores in the range between 52 and 60. This intermediate group has been labeled as “possibly alexithymic” (77). Our investigation was limited to non-alexithymic patients (who could have scores from 20 to 51) and alexithymic patients (who could have scores from 61 to 100) applying the criteria of Bagby and Taylor (33). In clinical practice it may be helpful to label patients as having or not having an attribute. Although categorization of continuous variables as in the case of alexithymia is quite common in clinical research it can go along with several serious drawbacks. Dichotomizing continuous variables can lead to a reduction in statistical power to detect relations with other variables (78). Moreover, dichotomization might increase the risk of positive results being false positives (79). Artificial dichotomization based on sample median poses the problem that various data-derived cut-points can be used in different studies so that their findings cannot be easily compared or processed in meta-analyses. The cut-off scores administered in our study to define alexithymia and non-alexithymia (33) have at least the advantage of being internationally recognized. Research results on the structure of the alexithymia construct have provided strong support that alexithymia is a dimensional construct. Taxometric statistical procedures produced unambiguously dimensional solutions, providing substantial evidence that the core alexithymia features are continuously distributed in the population (80, 81). Against this background, it is recommended to use dimensional analyses in future studies that examine the potential effect of the personality trait alexithymia on emotion perception in depression or other mental disorders.

A further limitation of our study is that explicit emotion identification ability of participants was not assessed. It is an interesting question whether the ability to explicitly identify and label facial emotions is related to performance in the face-in-the-crowd task which appears to measure primarily implicitly operating processes of categorization and comparison. It should be noted that when faces with intense expressions have been presented for longer durations or without time limit no impairments in emotion identification were found in alexithymic individuals (82–84). Although, we included the TMT-B to assess participants' general visual processing speed it is a limitation of our study that it did not comprise a non-social control condition requiring search for discrepancies between several complex stimuli. Thus, it remains unclear whether the observed alexithymia-related impairments are specific for social stimuli or represent general visual processing impairments. Future face-in-the-crowd research should administer complex non-social search tasks with multiple stimulus displays to enable stronger conclusions. These search tasks may consist of a texton or a non-texton target in a group of distractors (e.g., crosses, lines, or letters) that allow to assess processes of pre-attentive and attentive visual search for non-social stimuli (85, 86). A further important limitation of our investigation is that no healthy control group was included (neither non-alexithymic nor alexithymic healthy subjects). Therefore, it remains unclear whether one or both of our depressed patient groups show impairments in test performance or gaze behavior compared to healthy individuals. Future studies should investigate whether alexithymia in healthy persons is also associated with deficits in visual search for emotional faces. Finally, our study can be criticized for not having assessed patients' ratings of arousal and valence of the emotional faces presented in the experiment. However, when looking at the findings of several recent alexithymia studies on emotion face processing high alexithymia individuals' arousal and valence ratings of facial expressions did not differ from those of low alexithymia individuals (87–89). Thus, there is some evidence that intense facial expressions of basic emotions might be perceived as similarly arousing and positive (or negative) by highly alexithymic and non-alexithymic individuals.

Doubt has been expressed about the validity of self-report instruments assessing alexithymia, as such tests seem to depend on the abilities to monitor and report one's emotional states accurately (90). However, in the last 25 years, empirical studies have yielded considerable support for the reliability and validity of the TAS-20 (3). Moreover, in previous studies on alexithymia and emotion perception in which interview-based or observer-rated measures were administered in addition to self-report questionnaires self-reported alexithymia was found to be a better predictor of emotion processing than the scores derived from observer rating or interview (26, 28, 91).

In conclusion, the results from our eye-tracking study suggest that alexithymia goes along with impairments in visual processing of multiple facial emotions in clinical depression. According to the present findings, alexithymia is associated with prolonged scanning in the phase post-target detection in depression but might have no impact on the early phase of visual face processing. Thus, alexithymia seems not to be related to abnormalities in processes of attention guidance to discrepant emotional faces in clinical depression. The observed deficits could suggest difficulties in decision-making and/or target identification when processing multiple emotional facial expressions. Alexithymia might go along with a sense of uncertainty about the perceived expressions. Impairments of alexithymic depressed individuals in processing emotions in crowds of faces seem not limited to a specific affective valence. In group situations, depressed patients with alexithymia might be slowed in processing interindividual differences in emotional expressions compared with non-alexithymic depressed patients. This could be a disadvantage in comprehending non-verbal communication in groups. As alexithymia is quite common in depressed patients it appears advisable to control this personality characteristic in future research on emotion perception in clinical depression.

## Data Availability Statement

The raw data supporting the conclusions of this article will be made available by the authors, without undue reservation.

## Ethics Statement

The studies involving human participants were reviewed and approved by Ethics committee at the University of Leipzig, Medical Faculty. The patients/participants provided their written informed consent to participate in this study.

## Author Contributions

TS, AK, and CB conceived and designed the experiment. CB collected the data. TS, TH, VG, and AK outlined the manuscript. TS and CB analyzed the data. TS wrote the first draft of the manuscript. VG, TH, AK, and CB wrote sections of the manuscript. All authors contributed to the article and approved the submitted version.

## Conflict of Interest

The authors declare that the research was conducted in the absence of any commercial or financial relationships that could be construed as a potential conflict of interest.
