# Supplementary material for: Alexithymia Is Associated With Deficits in Visual Search for Emotional Faces in Clinical Depression
Source: Front Psychiatry. 2021 Jun 29;12:668019. doi: 10.3389/fpsyt.2021.668019 (PMC8275928; doi:10.3389/fpsyt.2021.668019)
Supplement: Supplementary file 1 [file Data_Sheet_1.docx]

**Supplementary material**

**Mean fixation time per distractor face before fixating the target**

A 6 (condition) x 2 (group) mixed ANOVA yielded a significant main effect of condition, *F*(5, 185) = 7.58, *p* < .001, $\eta_{p}^{2}$ = .17, but no main effect of group *F*(1, 37) = .45, *p* = .51, and no interaction effect *F*(5, 185) = .90, *p* = .48. Independent of study group, before target fixation participants fixated the distractor faces longest in the condition “angry target neutral distractors” and “neutral target angry distractors” followed by “happy target neutral distractors“ (see Table S1).

Supplementary Table S1: Mean fixation time (in milliseconds) per distractor face before fixating the target as a function of alexithymia and emotional quality of target and distractor face (means and SD (in brackets))

| Variable | | Alexithymic patients | Non-alexithymic  patients | |  |
| --- | --- | --- | --- | --- | --- |
| Fixation duration for **happy** distractors before fixating angry target | 671 (143) | | 670 (204) |  |  |
| Fixation duration for **neutral** distractors before fixating angry target  Fixation duration for **angry** distractors before fixating happy target | 856 (186)  729 (127) | | 780 (245)  719 (219) |  |  |
| Fixation duration for **neutral** distractors before fixating happy target | 794 (248) | | 761 (206) |  |  |
| Fixation duration for **angry** distractors before fixating neutral target | 769 (139) | | 798 (172) |  |  |
| Fixation duration for **happy** distractors before fixating neutral target | 698 (199) | | 609 (218) |  |  |
|  | |  |  |  |  |

**Mean fixation time on the target**

According to a 6 x 2 mixed ANOVA based on the duration of fixation on targets there was a significant effect of condition, *F*(5, 185) = 65.51, *p* < .001, $\eta_{p}^{2}$ = .64. No effect of group, *F*(1, 37) = 1.05, *p* = .31, and no interaction effect was found, *F*(5, 185) = 1.33, *p* = .25. Patients fixated the target face longest in the condition “neutral target angry distractors” followed by “angry target neutral distractors” (see Table S2).

Supplementary Table S2: Mean fixation time (in milliseconds) on target as a function of alexithymia and emotional quality of target and distractor face (means and SD (in brackets))

| Variable | | Alexithymic patients | Non-alexithymic  patients | |  |
| --- | --- | --- | --- | --- | --- |
| Fixation duration for **angry** target in happy distractors | 414 (76) | | 428 (87) |  |  |
| Fixation duration for **angry** target in neutral distractors  Fixation duration for **happy** target in angry distractors | 555 (128)  405 (75) | | 481 (103)  388 (61) |  |  |
| Fixation duration for **happy** target in neutral distractors | 393 (116) | | 374 (56) |  |  |
| Fixation duration for **neutral** target in angry distractors | 662 (173) | | 631 (122) |  |  |
| Fixation duration for **neutral** target in happy distractors | 461 (96) | | 434 (99) |  |  |
|  | |  |  |  |  |

**Mean fixation time per distractor face after fixating the target**

A 6 x 2 mixed ANOVA showed a main effect of condition, *F*(5, 185) = 30.43, *p* < .001, $\eta_{p}^{2}$ = .45, and a main effect of group, *F*(1, 37) = 4.87, *p* < .05, $\eta_{p}^{2}$ = .12. No interaction effect was found, *F*(5, 185) = .35, *p* = .88. After target fixation, alexithymic patients fixated distractor faces longer than non-alexithymic patients, regardless of face quality (see Table S3). In general, after fixation of the target, patients fixated the distractor faces by far longest in the condition “angry target neutral distractors” and “neutral target angry distractors” (see Table S3).

In addition, an ANCOVA was performed with level of education, reported depressive symptoms (BDI), antidepressant use, and sex as covariates. The results indicate that out of the covariates education level, *F*(1, 33) = 5.10, *p* ≤ .05, $\eta_{p}^{2}$ = .13, and depressive symptom severity, *F*(1, 33) = 5.79, *p* ≤ .05, $\eta_{p}^{2}$ = .15, had significant effects on the dependent variable. However, the effect of patient group remained significant, *F*(1, 33) = 4.14, *p* ≤ .05, $\eta_{p}^{2}$ = .11.

Supplementary Table S3: Mean fixation time (in milliseconds) per distractor face after fixating the target as a function of alexithymia and emotional quality of target and distractor face (means and SD (in brackets))

| Variable | | Alexithymic patients | Non-alexithymic  patients | |  |
| --- | --- | --- | --- | --- | --- |
| Fixation duration for **happy** distractors after fixating angry target | 256 (162) | | 143 (136) |  |  |
| Fixation duration for **neutral** distractors after fixating angry target  Fixation duration for **angry** distractors after fixating happy target | 631 (252)  303 (219) | | 496 (256)  235 (231) |  |  |
| Fixation duration for **neutral** distractors after fixating happy target | 347 (231) | | 233 (152) |  |  |
| Fixation duration for **angry** distractors after fixating neutral target | 615 (295) | | 472 (287) |  |  |
| Fixation duration for **happy** distractors after fixating neutral target | 291 (218) | | 229 (126) |  |  |
|  | |  |  |  |  |
